# Conservative Management of Postoperative Urinary Leak and Intra-Abdominal Abscess

**DOI:** 10.7759/cureus.40039

**Published:** 2023-06-06

**Authors:** Andrew B Herson, Camila A Villacreses, Gina M Rehm, Sean A Briceno, Kevin D Healey, Matthew R Brown, Brooke T Miller, Michael W Fountain

**Affiliations:** 1 Urology, Lake Erie College of Osteopathic Medicine, Jacksonville, USA; 2 Urology, Lake Erie College of Osteopathic Medicine, Fort Myers, USA; 3 Urology, Lake Erie College of Osteopathic Medicine, Tavares, USA; 4 Urology, Lake Erie College of Osteopathic Medicine, Bradenton, USA; 5 Urology, AdventHealth Waterman, Tavares, USA

**Keywords:** hematuria, genitourinary trauma, iatrogenic ureter injury, ureter leak, nephrostomy tube, ureteral injury

## Abstract

Ureteral injury is a rare occurrence in medical practice. Most cases encountered stem from blunt trauma or are iatrogenic, occurring during open abdominal or pelvic surgery and laparoscopic procedures. Prompt diagnosis of ureteral injury allows clinicians to avoid complications including ureteral strictures, abscess, renal failure, sepsis, and loss of the ipsilateral kidney. Treatment depends on whether the ureteral injury was discovered intraoperatively or if it was a delayed diagnosis. Several procedures can be used, including ureteroureterostomy, ureteroileal interposition, and nephrectomy. Stenting can also be a viable option as it can reestablish urinary drainage. Herein, we present the case of a 43-year-old male who presented with complaints of progressive abdominal pain that was subsequently diagnosed as a left ureteral injury and how the use of a ureteral stent allowed him to have a full recovery with optimized normal ureteral function.

## Introduction

Ureteral injury is a rare but serious risk of any pelvic or abdominal surgery and has the potential to cause serious complications such as sepsis, ureterovaginal fistula, renal failure, and loss of the ipsilateral kidney [1]. The ureter is protected from external and blunt trauma by its retroperitoneal location, diameter, mobility, and peritoneal coverage [2]. However, it is situated near various anatomical structures that are frequently involved in general and gynecologic surgeries [2]. Therefore, in reconstructive urology, ureteral repair is still challenging.

While the incidence of iatrogenic ureter injury ranges from 0.05% to 30%, depending on the skill level of the surgeon and the complexity of the procedure [3], iatrogenic injury accounts for 75% of ureteric injuries [4]. Gynecologic surgeries are the most common cause of iatrogenic ureteral injury [4]. The second most common cause is during general surgeries, with colorectal procedures being the most common subset [4,5]. Vascular and endoscopic urological procedures also pose a considerable threat to ureter injury, although a specific prevalence has not been documented [5]. Rates of iatrogenic ureteral injury have increased over recent decades due to the increase in laparoscopic approaches to surgery [4].

The preferred diagnosis is intraoperative, although most are delayed. Most injuries are diagnosed 48-72 hours post-op in an emergency setting [4]. Patients report symptoms including dysuria, hematuria, pyrexia, back or flank pain, a palpable mass, and signs of peritonitis in the setting of leukocytosis [4]. Diagnostic imaging typically includes ultrasound, triple-phase contrast-enhanced computed tomography (CT) urography, retrograde pyelogram, or intravenous (IV) urogram [3,4].

The distal third of the ureter is the most vulnerable to iatrogenic injury [3]. The middle and proximal thirds are less common [4]. Management options for ureter injuries range from endoscopic management to complex reconstructive surgery to auto-transplant, depending on the location [6].

Here, we present a case of ureteral injury and intra-abdominal abscess second to exploratory laparotomy/colostomy reversal with total rectal anastomosis and extensive adhesiolysis.

## Case presentation

A 43-year-old male with a past medical history of diverticulosis/diverticulitis and colon polyps presented to the emergency department (ED) complaining of abdominal pain that had gotten progressively worse over the last two days. Two days ago, the patient had an exploratory laparotomy/colostomy reversal with total rectal anastomosis and extensive adhesiolysis. Associated symptoms included abdominal cramping and diarrhea. The patient stated he had a fever of 101 degrees Fahrenheit at home. He denied any cough, chills, or chest pain.

On initial examination, the patient was tachycardic, and his abdomen was distended with diffuse moderate abdominal tenderness. Initial vital signs on presentation were as follows: temperature: 97.8 degrees Fahrenheit, heart rate: 128 beats per minute, respiratory rate: 18 breaths per minute, blood pressure: 106/76 millimeters of mercury (mmHg), and oxygen saturation of 92% in room air.

Laboratory tests included a complete blood count (CBC), a comprehensive metabolic panel (CMP), a urinalysis (UA), urine culture, lipase levels, troponin levels, and a CT scan of the abdomen and pelvis. The patient's lipase and troponin levels were within normal limits. The CBC revealed leukocytosis, normocytic normochromic anemia, and thrombocytosis (Table 1).

**Table 1:** Complete blood count WBC: white blood cell; RBC: red blood cell; L: Liter; g/dL: grams per deciliter; μm^3^: cubic micrometers; pg/cell: picograms per cell; Hb/cell: hemoglobin per cell; mm^3^: cubic millimeters; fL: femtoliters; NRBC: nucleated red blood cell count

	Laboratory Value	Reference Range
White blood cell count	16.97 × 10^9^/L	4.5 to 11.0 × 10^9^/L
Red blood cell count	4.24 x 10^12^/L	4.3-5.9 x 10^12^/L
Hemoglobin	25.5 g/dL	13.5-17.5 g/dL
Hematocrit	37.7%	41%-53%
Mean corpuscular volume	88.9 μm^3^	80-100 μm^3^
Mean corpuscular hemoglobin	29.5 pg/cell	25-35 pg/cell
Mean corpuscular hemoglobin concentration	33.2% Hb/cell	31%-36% Hb/cell
Red blood cell distribution width	12.8%	12-15%
Platelet count	850,000 mm^3^	150,000-400,000 mm^3^
Mean platelet volume	8.4 fL	7.5–11.5 fL
Nucleated red blood cell count, percentage (NRBC %)	0.0%	0%
Nucleated red blood cell count, absolute (NRBC, absolute)	0.00	0 nucleated RBC/100 WBC

The CMP revealed hyperglycemia and elevated alanine transaminase levels (Table 2).

**Table 2:** Comprehensive metabolic panel BUN: blood urea nitrogen; eGFR: estimated glomerular filtration rate; A/G ratio: albumin to globulin ratio; mg/dL: milligrams per deciliter; mL/min/1.73m^2^: milliliters per minute per 1.73 meters squared; mmol/L: millimoles per liter; g/dL: grams per deciliter; U/L: units per liter

	Laboratory Value	Reference Range
Sodium	138 mmol/L	135-145 mmol/L
Potassium	4.5 mmol/L	3.5-5 mmol/L
Chloride	99 mmol/L	95-105 mmol/L
Carbon dioxide	27 mmol/L	22-32 mmol/L
Anion gap	11 mmol/L	4 to 12 mmol/L
BUN	17 mg/dL	7-25 mg/dL
Creatinine	1.2 mg/dL	0.7-1.5 mg/dL
BUN/Creatinine ratio	14.2	6-22
Glucose	129 mg/dL	70-100 mg/dL
Calcium	9.2 mg/dL	8.5-10.5 mg/dL
Aspartate transaminase	26 U/L	10-35 U/L
Alanine transaminase	39 U/L	0-31 U/L
Alkaline phosphatase	99 U/L	25-125 U/L
Protein, total	7.0 g/dL	6.5-8.1 g/dL
Albumin	3.80 g/dL	3.5-5.0 g/dL
Globulin	3.2 g/dL	2.0-3.5 g/dL
A/G ratio	1.2 g/dL	0.8-2.0 g/dL
Bilirubin, total	0.5 mg/dL	0.0-1.2 mg/dL
eGFR	77 mL/min/1.73m^2^	>60 mL/min/1.73m^2^

The patient’s urinalysis showed hematuria (Table 3).

**Table 3:** Urinalysis pH: potential hydrogen

	Laboratory Value	Reference Range
Color, urine	Yellow	Yellow
Clarity, urine	Clear	Clear
Glucose qualitative, urine	Negative	Negative
Protein qualitative, urine	Negative	Negative
Bilirubin, urine	Negative	Negative
Urobilinogen, urine	Negative	Negative
PH, urine	5.0	5.0-7.0
Blood, urine	2+	Negative
Ketones, urine	Negative	Negative
Nitrite, urine	Negative	Negative
Leukocyte esterase, urine	Negative	Negative
Specific gravity, urine	1.015	1.005-1.030

**Figure 1:**
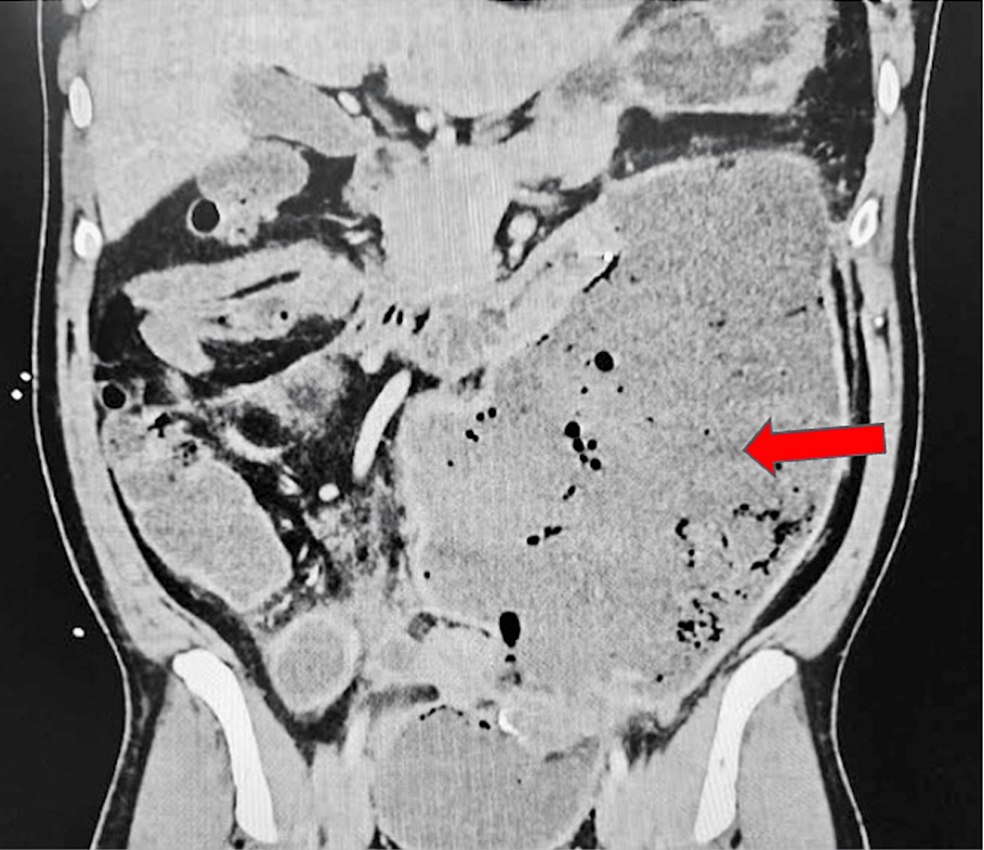
CT with contrast of abdomen and pelvis CT with contrast of the abdomen and pelvis demonstrated a large fluid and gas collection centered in the left abdomen (red arrow), which measured approximately 18 x 9.5 centimeters at its widest margins and extended approximately 26 centimeters craniocaudally.

An abdominal and pelvic CT was ordered due to worsening abdominal pain two days postoperatively (Figure 1).

The patient was diagnosed with a post-procedure intra-abdominal abscess and sepsis without acute organ dysfunction due to an unspecified organism. He was then admitted to the inpatient service and started on IV piperacillin/tazobactam. Urine cultures were pending. Interventional radiology was consulted, and the patient underwent percutaneous abscess drainage. The following day, repeat abdominal and pelvic CTs revealed subtotal abscess drainage. Laboratory analysis of the abscess fluid revealed a significant elevation of creatinine compared to serum levels, indicating the presence of urine (Table 4). 

**Table 4:** Abscess fluid creatinine levels mg/dL: milligrams per deciliter

	Laboratory Value	Reference Range
Creatinine, fluid	30.01 mg/dL	None

Bacterial growth was detected in both the urine and abscess fluid cultures (Table 5, 6).

**Table 5:** Urine culture CFU/mL: colony forming unit per milliliter

	Laboratory Value	Reference Range
Urine culture	10,000 CFU/mL of Escherichia coli	none
	10,000 CFU/mL Klebsiella pneumoniae	none

**Table 6:** Abscess fluid culture

	Laboratory Value	Reference Range
Abscess fluid culture	Moderate growth of Escherichia coli	none
	Moderate growth of Klebsiella pneumoniae	none
	Light growth of Candida dubliniensis	none

**Figure 2:**
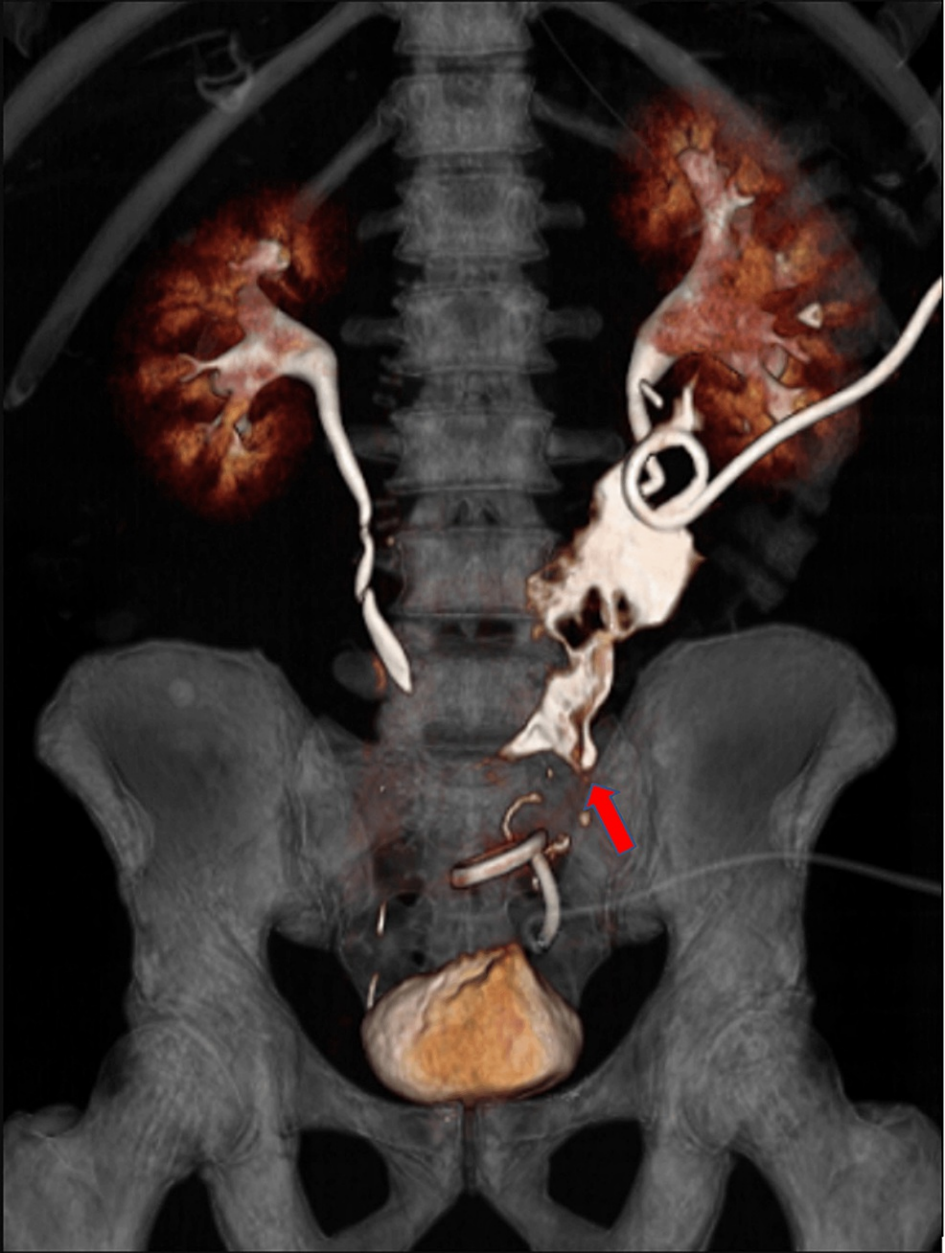
Computerized tomography urogram A virtual reality reconstructed CT urogram shows the escape of contrast material (red arrow) into the area of the previously drained fluid collection.

A computerized tomography urogram (CTU) revealed contrast extravasation from the left midureter, indicative of an active urinary leak from the left midureter just above the colonic anastomosis (Figure 2).

**Figure 3:**
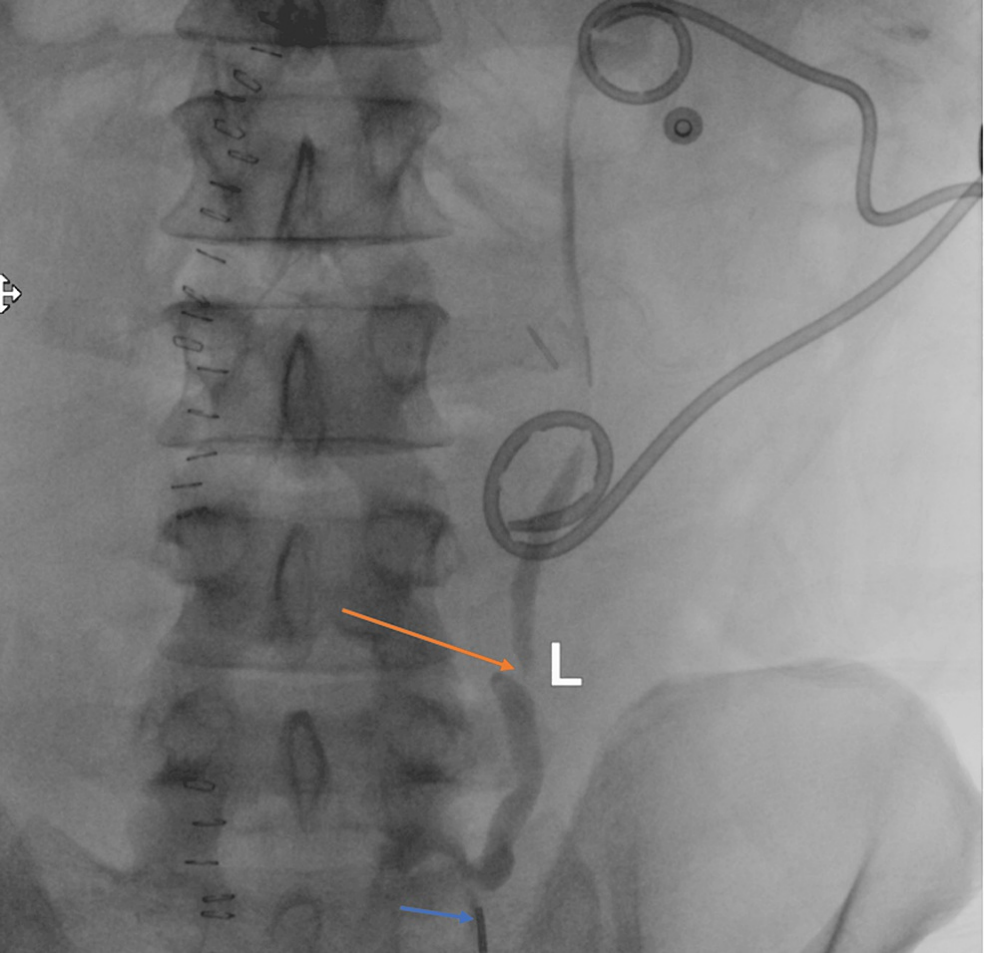
Ureteral disruption Fluoroscopic image showing an antegrade pyelogram stopping abruptly at the level of the left laterality marker (orange arrow). Simultaneously, a retrograde ureterogram shows complete obstruction at the level of the left laterality marker as introduced through the ureteral catheter visible at the bottom of the image (blue arrow).

**Figure 4:**
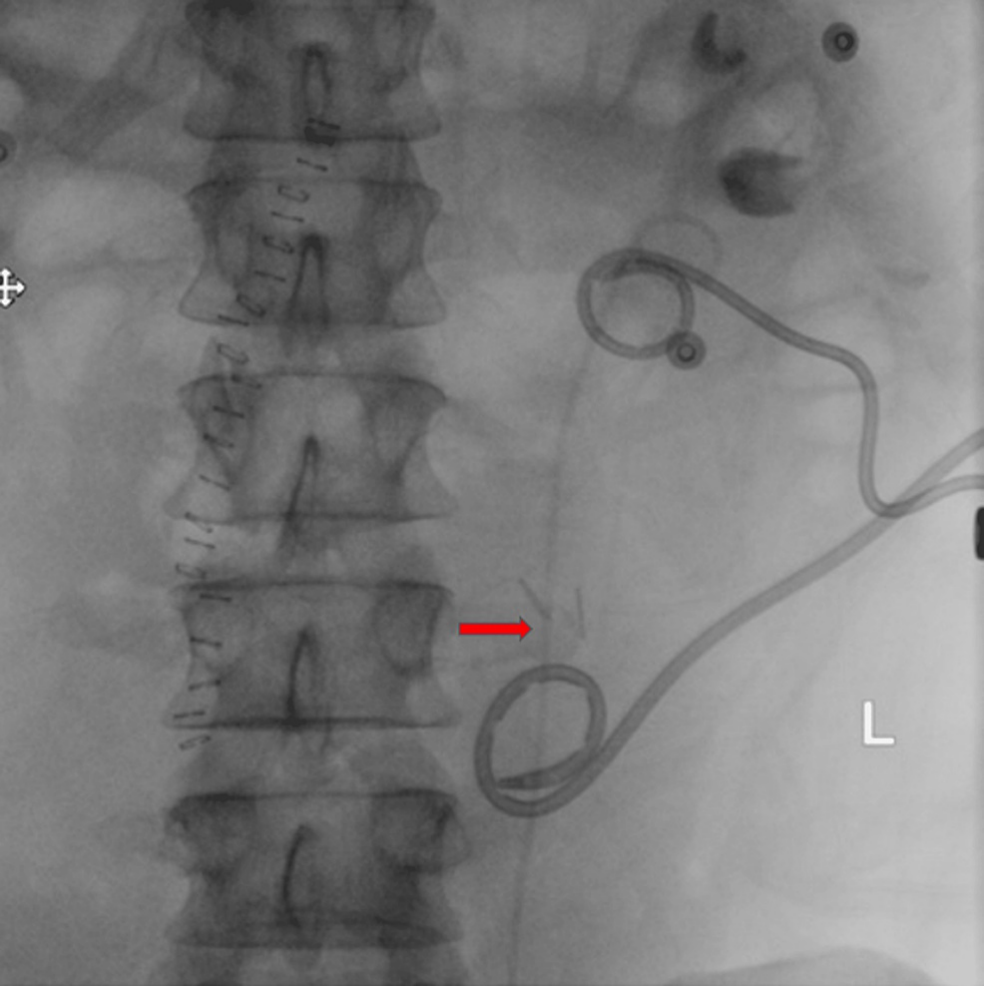
Ureteral stent Fluoroscopic image showing successful placement of a ureteral stent (red arrow).

Interventional radiology placed a left nephrostomy tube. Urology placed a ureteral stent under ureteroscopic guidance (Figure 3, 4).

**Figure 5:**
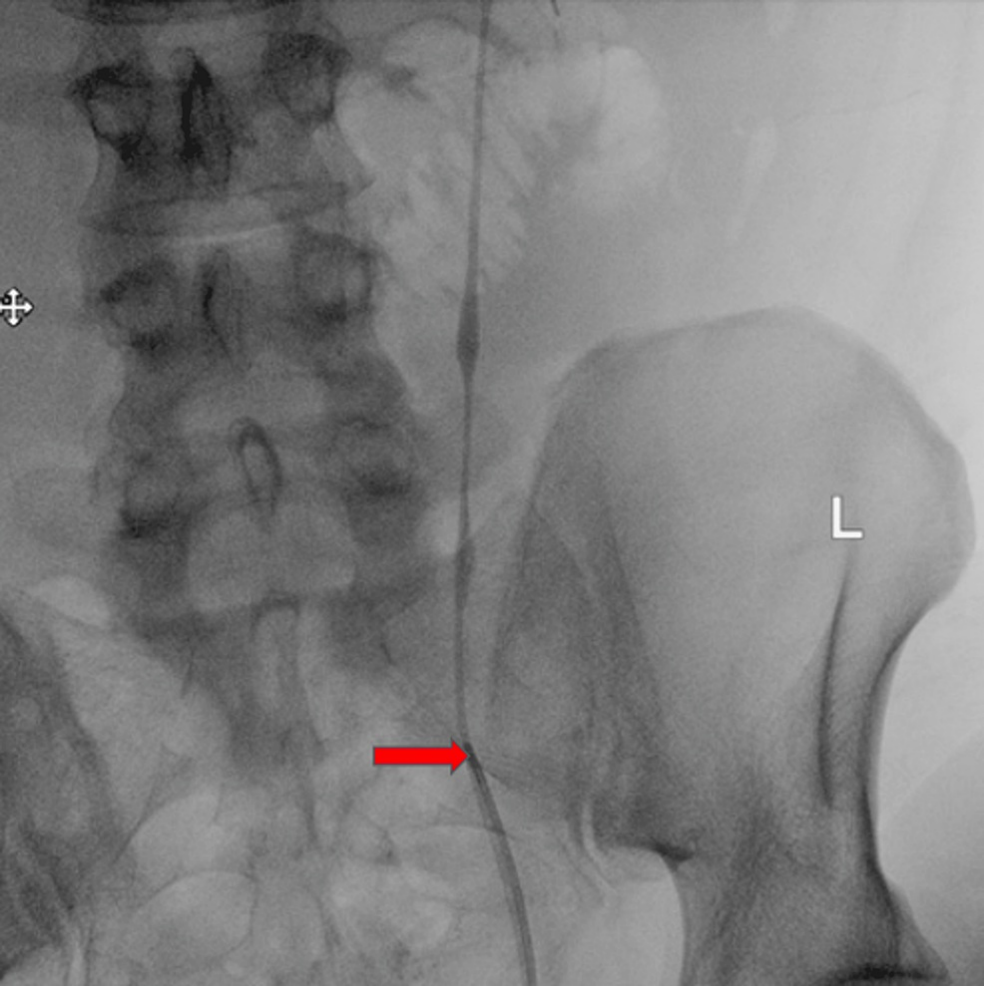
Restored ureteral patency The fluoroscopic image shows the ureteral catheter (red arrow) injecting contrast uninterrupted through the length of the ureter, reaching the pelvis and calyces.

The patient was discharged with a 10-day course of ciprofloxacin and was encouraged to follow-up regarding the removal of the drain and stent. At the one-month follow-up, the nephrostomy tube was removed. One month later, the patient underwent a cystoscopy-guided removal of the ureter stent. During this procedure, after the stent was removed, a retrograde pyelogram was performed, demonstrating successful treatment with visualization of an intact left ureter and renal pelvis (Figure 5).

## Discussion

A ureteral injury is a rare occurrence. It comprises less than 1% of blunt and penetrating genitourinary trauma. However, it does pose a serious complication of iatrogenic trauma that occurs during open surgery, laparoscopic surgery, or endoscopic procedures [7]. This trauma can lead to complications such as ureterovaginal fistula, abscess, ureteral strictures, urinoma, intra-abdominal sepsis, renal failure, loss of the ipsilateral kidney, and even death if they go unrecognized or mismanaged [1,7].

The ureters are in the retroperitoneum and serve to transport urine from the kidneys to the bladder. They are composed of three segments. The proximal segment is part of the ureter that extends from the ureteropelvic junction (UPJ) to the area where the ureter crosses the sacroiliac joint. The middle segment courses along the bony pelvis and iliac vessels. The segment that comprises the pelvic or distal ureter extends from the iliac vessels to the bladder [7,8]. As such, ureteral injuries can be classified based on the location of the injury as well as when the diagnosis is made (immediate vs. delayed diagnosis).

The American Association for the Surgery of Trauma (AAST) uses a grading classification for these injuries, with Grade I described as a contusion or hematoma without devascularization, the highest grade, and Grade V involving laceration with avulsion with greater than 2 cm of devascularization [5,7]. Most iatrogenic ureteral injuries involve the pelvic ureter. The ureter is at risk due to its location near the uterine and gonadal vessels, iliac arteries, sigmoid and inferior mesenteric vessels, colon, and rectum [8].

Since the early 1990s, the incidence of iatrogenic ureteral injury has increased by more than 7.5-fold as a result of the rise in laparoscopic and robotic procedures [9]. Due to this, the prevention of ureteral injury has gained a keen interest in recent years. One of the proposed preventions has been preoperative ureteral catheterization to aid in identifying the ureters and avoid iatrogenic injury. However, some studies have indicated that preoperative stenting has increased the incidence of ureteral injury [5,9].

Due to ureteral injuries often being subtle, clinicians must have a high index of suspicion for them. Delayed diagnosis can present as flank or abdominal pain that is persistent, a flank mass, prolonged ileus, urinary tract infection, or hydronephrosis with an accompanying elevated creatinine and blood urea nitrogen (BUN). Hematuria can also be present, but the absence of hematuria does not exclude injury [7]. Ideally, an iatrogenic injury would be identified in the intraoperative setting and subsequently dealt with immediately. However, approximately 50-70% of ureteral injuries are not diagnosed in the acute setting [5]. In the intraoperative setting, the most sensitive diagnostic study is the use of a retrograde pyelogram, which evaluates the location and extent of the ureteral injury [3,5,7]. A diagnosis can also be performed with an anterograde pyelogram if the patient has antegrade access. A CT urogram can also be utilized to identify any form of ureteral injury. Suspicion of injury should be high if CT imaging studies indicate perinephric stranding, low-density fluid around the kidney and ureters, perinephric hematoma, dilation of the ureter or deviation, contrast extravasation, or a urinoma [3,4,7]. An intravenous urogram (IVU) shows contrast extravasation and a non-opaque distal ureter [3]. Ultrasound is frequently used in emergency settings. The presence of ascites, hydronephrosis, or absent ureteric jets is suggestive of ureter injury [3]. When a ureteral injury is identified in a delayed setting, ureteral strictures are often formed. As such, it is suggested that when ureteral injuries are discovered in this manner, they should be immediately repaired within 72 hours of detection.

Treatment approaches depend on the patient's overall health and the location of the injury. Surgery should be considered a last resort, as repeated surgeries can be detrimental to an unstable patient [1]. Injuries above the pelvic brim can be addressed using several procedures, including ureteroureterostomy, ureteroileal interposition, and nephrectomy [10]. Ureteroureterostomy is the preferred procedure for treating uncomplicated injuries to the upper and middle third of the ureter [11]. For injuries occurring below the pelvic brim, a ureteroneocystostomy with a psoas hitch or Boari bladder flap is recommended [11]. For patients with an anatomical defect of the ureter that cannot be treated surgically or those with a poor general state, permanent transrenal ureteral diversion may be a viable treatment option [1]. Ureteral stenting is a minimally invasive approach to reestablish urinary drainage [11]. While stenting may be effective for injuries resulting in shorter strictures, it has limited long-term success rates in cases of longer strictures, and surgical reconstruction is necessary to achieve successful outcomes [11]. Although injuries to the upper and middle third portions of the ureter are usually treated with ureteroureterostomy, in this case, ureteral stenting provided urinary diversion while giving time for the leak to heal, which led to satisfactory outcomes. This highlights the continued role of conservative management in these complex cases.

## Conclusions

Although an iatrogenic ureteral injury is a rare complication, it can have severe consequences for patients undergoing surgical procedures. Important steps should be taken to protect the ureters preoperatively. The diagnosis of ureteral lacerations requires a high index of clinical suspicion. Prompt diagnosis is paramount to preventing long-term complications. If detected early, surgery is the preferred method for treating ureteral injuries. However, conservative methods like ureteral stenting should still be considered. With the implementation of pre-operative stenting, different surgical techniques, and new equipment, we hope to see a downward trend in the cases of ureteral injury following procedures in the future.
